# Strengthening pill press control to combat fentanyl: Legislative and law enforcement imperatives

**DOI:** 10.1016/j.rcsop.2023.100321

**Published:** 2023-08-22

**Authors:** Nicholas Lassi

**Affiliations:** Guanghua Law School, Zhejiang University, China

**Keywords:** Fentanyl crisis, Counterfeit pharmaceuticals, Pill press control, Global drug governance, United States, China, Mexico

## Abstract

**Background:**

Fentanyl has garnered significant focus from governments, academics, and the media due to the unparalleled increase in overdose fatalities it has triggered in North America. However, the pill presses, dies, and molds used to tablet counterfeit fentanyl-laced pills in Mexico and the United States (U.S.) have gone largely unstudied despite their substantial influence on fentanyl production. The Biden-Harris administration's recent initiative to intensify the prosecution of illicit fentanyl supply chains and the U.S. Treasury Department's decision to impose sanctions on numerous Chinese entities for illicit pill press production and distribution highlights the need to increase controls on pill-press proliferation.

**Objective:**

Review the existing legislative frameworks and enforcement in the U.S., China, and Mexico that pertain to pill press control and provide recommendations for improvement.

**Methods:**

A literature review was conducted to gather information on counterfeit fentanyl-laced pill production, pill press control, and related legislative frameworks and enforcement in the U.S., China, and Mexico. Federal and state laws, policies, and enforcement mechanisms were reviewed to identify gaps, limitations, and potential for improvement. A comparative study was then performed to determine the strengths and weaknesses of these states' prevailing legislative structures and enforcement mechanisms.

**Results:**

In the U.S., pill press laws at the federal level are limited, and state laws are generally weak or nonexistent. The U.S. Controlled Substances Act should be amended to include harsher penalties for the unregistered possession of pill presses with or without the intent to tablet illicit substances. China, the primary source of illicitly distributed pill presses, does not have laws regulating pill presses and should enact strong pill press legislation and engage in rigorous enforcement.

**Conclusions:**

As pill presses are integral to the final and crucial step in counterfeit fentanyl-laced pill production, pill press control should receive more legislative and law enforcement attention in the U.S., China, and Mexico.

## 1 Introduction

**Fig. 1:**
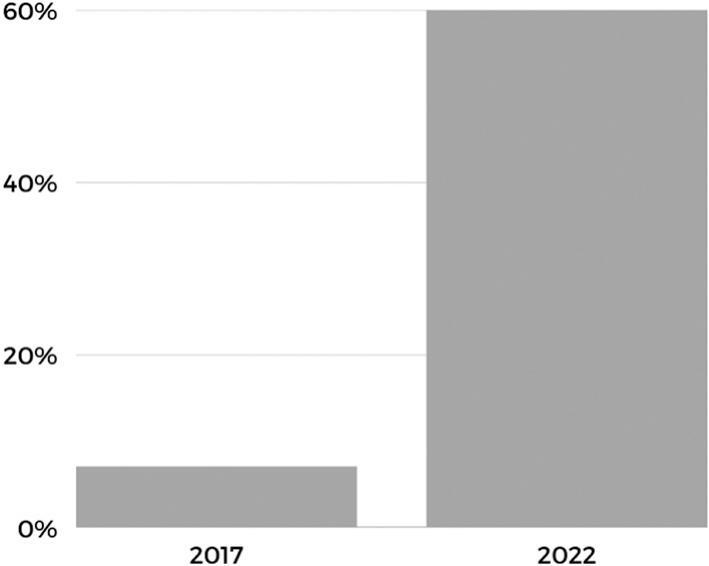
Rise in counterfeit pills containing potentially lethal doses of fentanyl.^1^^,^^2^

The threat posed by counterfeit fentanyl-laced pills to public health in North America is escalating. The percentage of counterfeit fentanyl-laced pills containing potentially deadly amounts of fentanyl has increased dramatically in recent years. In 2017, 7 % of counterfeit pills contained potentially lethal doses of fentanyl.^1^ By 2022, that number had risen to a startling 60% (Fig. 1).^2^ This trend should be cause for alarm, especially considering that the U.S. Drug Enforcement Administration (DEA) confiscated 50.6 million fentanyl-laced counterfeit pills in 2022, double the amount seized the previous year.^2^ These counterfeit pills are designed to look like legitimate pharmaceuticals, such as Vicodin® and Oxycontin® (commonly known as “blues” or “M30s”), but contain only fentanyl and different fillers.^3^ These counterfeits are typically tableted in Mexico or the U.S. using Chinese-made pill presses,^4^^,^^5^ with fentanyl synthesized in Mexico using precursor chemicals sourced from China.^6^^,^^7^

In response to the jarring rise in fentanyl-related overdose deaths in the U.S., the Biden-Harris administration launched an initiative in April 2023 to combat illicit fentanyl supply chains.^8^ This initiative focuses explicitly on controlling pill presses and their components, such as dies and molds that shape powder fentanyl and filler materials into counterfeit pills. In June 2023, the U.S. Treasury Department imposed sanctions on numerous Chinese entities for allegedly distributing pill presses and other equipment used to manufacture illicit fentanyl in Mexico and the U.S.^5^ Given that China has been accused of manufacturing and distributing pill presses that are illegally imported into North America, and that China supplies much of the chemical precursors used to produce fentanyl and its analogues,^4^^,^^7^^,^^9^^,^^10^ it should play a key role in controlling the spread of pill presses.

Central to addressing the challenges posed by fentanyl is the implementation of comprehensive harm reduction, treatment, drug education, and public awareness initiatives within the U.S. These measures collectively form the cornerstone for mitigating the suffering and destruction brought about by fentanyl. Simultaneously, considering the exceptionally lethal nature of fentanyl and its various analogues, efforts to interrupt supply chains and production processes are valuable. By taking steps to disrupt the influx of these substances into North America and other global destinations, the potential to safeguard lives is increased and the risk of exacerbating public health emergencies on a larger scale is reduced. This two-pronged approach, emphasizing both domestic interventions and international supply chain and production control, serves as a more holistic strategy to combat the threats posed by fentanyl and its derivatives.

The regulatory framework for pill presses remains limited in the U.S., China, and Mexico, enabling bad actors to manufacture, distribute, possess, and use unregistered pill presses with little risk. This study addresses the challenges of regulating pill presses used to produce counterfeit fentanyl-laced pills. Existing U.S., Chinese, and Mexican pill press laws and means of enforcement are reviewed to determine why legislation among these states has been ineffective. The weaknesses of state and federal laws in the U.S. and national laws in China and Mexico for pill press control are explained, and recommendations and strategies for improvement are provided.

## 2 Methods

This study addressed the challenges of regulating pill presses producing counterfeit fentanyl-laced pills. A comprehensive survey of the literature was undertaken to gather insights on the production of counterfeit fentanyl-laced pills, the regulation of pill presses, and associated legislative frameworks and enforcement practices within the U.S., China, and Mexico. Federal and state-level regulations, policies, and means of enforcement were examined to identify inconsistencies, limitations, and possibilities for improvement. The materials and data reviewed were collected from Google Scholar, LexisNexis, international organizations, legislative documents, and media reports. Pill press law from several international regulatory bodies, states, and institutions was reviewed, including U.S. law from the Legal Information Institute at Cornell Law School and the U.S. House of Representatives Office of the Law Revision Counsel (OLRC), Chinese law from the General Administration of Customs and Peking University's Chinalawinfo Database, and international treaty provisions from the United Nations Office on Drugs and Crime (UNODC). Regulatory guidelines from various official bodies were reviewed, such as those from the International Narcotics Control Board (INCB), the U.S. Drug Enforcement Administration (DEA), and the U.S. Department of Justice (DOJ). Media outlets such as CNN, Reuters, Al Jazeera, and the Associated Press provided additional insight. The resources selected for this study were chosen based on their relevance, reliability, publication date, and ability to provide comprehensive information on the control and use of pill presses. IRB and/or ethics board approval for this study was not applicable.

## 3 Results

Counterfeit fentanyl-laced pills are primarily manufactured in Mexico by the Sinaloa and Jalisco Cartels (Cártel de Jalisco Nueva Generación or CJNG) and then smuggled into the U.S.^6^^,^^7^ According to Mexican President Andres Manuel Lopez Obrador, Mexican law enforcement identified approximately 1400 illegal fentanyl labs in 2022, indicating that a considerable amount of fentanyl is being synthesized in Mexico.^11^ These criminal organizations use cheap fentanyl synthesized in Mexico and bulking agents, dies, molds, and pill presses to produce counterfeit fentanyl-laced pills. The equipment costs are relatively low (around $1000 for an average pill press and its various parts), and the chemicals are inexpensive (fentanyl is produced using low-cost chemical precursors).^12^ The potential return on costs from street-level sales is enormous,^13^ leading to increased distribution and use of illicit pill presses and the availability of counterfeit fentanyl-laced pills. This has resulted in an urgent need for strong legislation, increased law enforcement efforts, and international cooperation to curb the distribution of illegal pill presses and molds.

Illicit fentanyl manufacturers and distributors utilize a range of pill press dies and molds to replicate legitimate pharmaceuticals, enabling the production of counterfeit fentanyl-laced pills in various forms. This tactic deceives unsuspecting users into consuming what they believe are diverted prescription drugs. Also, these dies and molds often feature new and pop-culture-based pill face designs and come in various colors,^14^ enticing younger people to knowingly or unknowingly consume fentanyl-based products. Ultimately, pill presses facilitate marketing mechanisms that hook new generations on deadly fentanyl.

Determining the appropriate fentanyl dosage for counterfeit pills is left to the discretion of illicit drug manufacturers and criminal organizations. This practice results in a significant variance in the amount of fentanyl contained within counterfeit pills. The DEA has seized imitation tablets containing as little as 0.02 mg and as much as 5.1 mg of fentanyl per tablet.^15^ Fentanyl is so potent that as little as 2.0 mg, equivalent in size to the graphite tip of a pencil, can be a potentially lethal dose. In contrast, the DEA considers a lethal dose of heroin to be around 30 mg. As a result of this lethality, record numbers of Americans are dying from fentanyl, fentanyl analogues, and fentanyl-based polydrugs.^16^^,^^17^ In the twelve months from May 2020 to April 2021, the U.S. reported a record-breaking 100,306 overdose deaths, of which 75,673 were opioid-related (primarily fentanyl), representing a 28.5% increase from the previous year.^18^ In 2022, there were approximately 107,081 overdose deaths, and over two-thirds were a consequence of fentanyl or other illicit synthetic opioids.^19^

Because of the high variability of fentanyl content in counterfeit pills, users are often unaware of how much fentanyl they consume. Furthermore, as mentioned, many users unknowingly ingest fentanyl-laced counterfeit pills, believing they are consuming diverted prescription opioids that are generally less potent than fentanyl. This leads to dangerous situations where users increase their consumption or combine the counterfeit pills with other drugs, not realizing the potentially lethal consequences of such actions. Taken together, highly variable fentanyl content, unknowing consumption, and mixing in other hard narcotics are exceptionally hazardous.

The production of illicit fentanyl in Mexico heavily relies on precursor chemicals and pill presses sourced from China.^5^^,^^7^ China's class-wide scheduling of fentanyl and its analogues in 2019 made producing and distributing finished fentanyl and its analogues illegal.^20^ However, this has not fully addressed the fentanyl issue. Chinese chemical manufacturers modify the chemical structures of prohibited fentanyl precursor substances to develop new and unscheduled analogues. They also produce other fentanyl precursor chemicals that have yet to be scheduled. These Chinese chemical companies then export the legal or weakly regulated fentanyl precursor chemicals to Mexico and India for the final syntheses to complete the production process.^7^ Most illegal pill presses used in Mexico and the U.S. during the final stages of this process are manufactured in China.^4^^,^^5^ This roundabout method allows the illicit fentanyl trade to continue unimpeded, exacerbating North America's extensive public health crises.

As a result, Mexican drug cartels, who have long been connected to Chinese chemical manufacturers and cartels in the illicit drug trade,^6^^,^^7^ have seamlessly integrated Chinese-made pill presses and fentanyl precursors into their production and distribution networks. This integration has enabled Mexican drug cartels to produce counterfeit fentanyl-laced pills on an industrial scale. Under Mexican federal law, importing, exporting, and possessing pill presses is prohibited without annually reporting these activities to the Mexican Ministry of Economy. Mexico's Attorney General of the Republic must also be made aware of any attempts to divert presses to alternative destinations.^21^ These activities are rarely reported to the Mexican Ministry of Economy or prosecuted in Mexico.

U.S. port and customs enforcement of illicit pill press importation remains challenging, predominantly due to the sheer amount of goods processed at ports of entry each day. A new shipping container arrives at a U.S. port every 7.8 s.^4^ Transnational criminal organizations (TCOs) are also skilled at concealing pill presses to bypass port authorities. In China, pill press traffickers circumvent U.S. regulations by mislabeling presses or breaking them down into individual parts that are mislabeled as legitimate products, such as “furniture parts” or “machine spare parts.”^10^^,^^22^ These parts are difficult to identify and are often waved through customs, only to be reassembled as unregistered pill presses at their destination and used for illicit purposes. As the demand for illicit pill presses continues to rise, confiscations by U.S. officials at ports of entry and fentanyl production sites have increased. For instance, pill press confiscations at International Mail Facilities in the U.S. have risen 19-fold from 2011 to 2017.^4^^,^^23^

## 4 Discussion

### 4.1 U.S. pill press laws and enforcement

The importation of pill presses into the U.S. is subject to authorization from the DEA. The DEA and the U.S. Customs and Border Protection (CBP) are sanctioned to confiscate unregistered pill presses when captured. Federal law prohibits the sale, possession, and use of unregistered pill presses, and obtaining a DEA registration number is mandatory to possess a pill press and manufacture drugs. However, the registration process heavily relies on self-reporting, typically during the sale and transfer of machines. The Controlled Substances Act (CSA) mandates that anyone involved in the transaction of a pill press must maintain records of the event for two years, which should include the transaction date, the identity of each party, a description of the machine, and means of transference; a report of the transaction containing this information should be provided to the Attorney General (21 U.S.C. § 830).^24^ Domestic transfers also require a verbal notification to the DEA by the transferring parties. Nevertheless, self-reported registrations are challenging to regulate, resulting in many presses going unregistered. Also, criminals ignore these provisions with little concern as they typically engage in other criminal activity with more significant penalties attached.

Notably, the CSA's penalties for possessing an unregistered pill press with the intent to manufacture counterfeit fentanyl-laced pills are relatively light, wherein:

To possess any three-neck round-bottom flask, tableting machine, encapsulating machine, or gelatin capsule, or any equipment, chemical, product, or material which may be used to manufacture a controlled substance or listed chemical, knowing, intending, or having reasonable cause to believe, that it will be used to manufacture a controlled substance or listed chemical….shall be sentenced to a term of imprisonment of not more than 4 years, a fine under title 18, or both…. (21 U.S.C. § 843, (a)6, (d)1).^25^

The same penalties are stipulated for dies, molds, and stones for imprinting under 21 U.S.C. § 843, (a)5, (d)1. If the tableting involves trademark infringement, such as stamping a pill with the trademarked image or symbol of a legitimate pharmaceutical, the penalties can be increased. Under 18 U.S.C. § 2320, (a)(4), (b)(1), whoever intentionally traffics a drug and knowingly uses a counterfeit mark on or in connection with such drug, or attempts or conspires to do so can be fined up to $2,000,000 or imprisoned not more than ten years, or both. Violators of trademark laws can also be prosecuted under the Commerce and Trade Code, under which any goods sold using a counterfeit trademark can be confiscated by court order (15 U.S.C. § 1116, d(1)(A)).^26^ However, more substantial penalties for pill press possession with the intent to produce counterfeit and non-counterfeit fentanyl-laced pills are necessary to combat the enduring fentanyl epidemic.

The CSA explicitly imposes harsher penalties for possessing a pill press with the intent to manufacture tablets containing methamphetamine, with a maximum term of imprisonment of ten years. The CSA does not set the same severe penalties for the possession of pill presses with the intent to manufacture tablets containing fentanyl. This disparity is especially concerning given that fentanyl is more dangerous than methamphetamine in terms of death rates. The enhanced penalties for possessing a pill press with the intent to produce methamphetamine includes: “Any person who, with the intent to manufacture or to facilitate the manufacture of methamphetamine, violates paragraph (6) or (7) of subsection (a), shall be sentenced to a term of imprisonment of not more than 10 years…” (21 U.S.C. § 843, (d)2).^25^ Only methamphetamine is subject to these advanced penalties.

The penalties for pill press possession with the intent to manufacture narcotics vary widely among U.S. states; in many cases, the penalties are weak or nonexistent. According to a 2021 study by the Legislative Analysis and Public Policy Association (LAPPA), six states, including Maine, Minnesota, Massachusetts, Missouri, Kentucky, and Vermont, have no prohibitions on pill presses or molds, while the majority of states employ relatively weak regulations derived from the Food, Drug, and Cosmetic Act.^1^ Under this Act, first-time pill press violators are subject to penalties of up to one-year imprisonment, a fine extending to $1000, or both. Subsequent violations increase this punishment to three years imprisonment, a fine extending to $10,000, or both (21 U.S.C. § 333).^27^ Therefore, state laws require significant improvements to tackle the challenges posed by fentanyl.

Florida's 2019 pill press laws could serve as a model for improved legislation, as they prohibit individuals from possessing, purchasing, delivering, selling, or possessing with intent to sell or deliver a tableting machine or controlled substance counterfeiting materials (Fla. Stat. 2022, § 893.147, (7)a).^28^ Violators commit a third-degree felony. Furthermore, individuals violating this provision knowing that their actions will result in the unlawful manufacture of a controlled substance containing fentanyl, fentanyl-related substances, or fentanyl analogues commit a second-degree felony (Fla. Stat. 2022, § 893.147, (7)2).^28^ Such violators face imprisonment for up to 15 years, a fine of up to $15,000, or both (Fla. Stat. 2022 § 893.147(7)(d)).^28^ This approach will help clarify and strengthen the penalties for pill press possession with or without the intent to manufacture fentanyl and fentanyl-related substances.

In March 2023, U.S. Representative David Kustoff reintroduced the Criminalizing Abused Substances Templates (CAST) Act in the House of Representatives, which aims to amend the Controlled Substances Act (CSA) to enhance the federal penalty for possessing a pill press with the intent to tablet fentanyl.^29^ This Act extends the maximum sentence for this crime to 20 years imprisonment and would improve the CSA's ability to address the growing fentanyl crisis in the U.S.

### 4.2 Chinese pill press laws and enforcement

Despite the illegality surrounding pill presses in many countries, China has yet to enact laws regulating the production, sales, or exportation of pill presses, dies, molds, or other associated parts.^30^ However, pill press smugglers in China must contend with foreign authorities on the receiving end of these transfers, leading them to engage in deceptive practices such as dismantling machines and mislabeling individual parts for transportation. While these tactics help evade foreign authorities, they leave smugglers open to prosecution by China's General Administration of Customs (GAC). The GAC examines contracts, records, accounts, and other documents connected to goods and articles' inward and outward transport and intercepts those items deemed to violate Customs Law (Article 5).^31^ Under Article 84 of the Customs Law of the People's Republic of China, anyone who “counterfeits, falsifies, purchases, or sells Customs documents” can be investigated for criminal liability.^31^ Article 86 details the limited monetary fines for making false declarations to Customs.^31^ Given the severity of the narcotics-related materials involved, these provisions and accompanying fines are relatively weak, and meaningful prosecution is easily circumvented by providing false return addresses and sender information. In the coming decade, China must decide on the degree of regulatory measures, supervision, and enforcement necessary to combat the illicit international distribution of pill presses. Resistance from local governments, corporate interests, and China's tendency to avoid controversial or economically injurious regulations may hinder the process of legal and enforcement restructuring.

As a signatory to the 1988 United Nations Convention against Illicit Traffic in Narcotic Drugs and Psychotropic Substances, China is obligated to prevent the proliferation of illicit pill presses and consider the impact of its policies on other countries. According to Article 13 of the Convention, governments must take appropriate measures to prevent the trade and diversion of materials and equipment used for the illicit production of narcotics and psychotropic substances and cooperate with other states in this control.^32^ Similarly, Article 3 provides the basis for countries to prohibit the manufacture, transport, or distribution of equipment with the knowledge that it will be used for the cultivation, production, or manufacture of illicit substances. This Convention also forbids the possession of such equipment and materials (1(a)(iv) and 1(c)(ii)).^32^ These articles provide valuable guidance for governments seeking to combat the proliferation of illicit pill presses.

China should enact clear and stringent rules governing pill press production, distribution, possession, and export. Comprehensive oversight and enforcement mechanisms, including increased monitoring of online marketplaces, pill press manufacturers and distributors, and ports of exit, should accompany these regulations. Such measures should entail robust penalties, such as substantial fines and incarceration, to improve compliance.

The Chinese government should establish a comprehensive regulatory system to monitor and trace the country's production, distribution, and possession of pill presses. This system should encompass all types of pill presses, including manual or hand-powered, semiautomatic, and industrial machines, and the associated dies, molds, plates, or other materials used to imprint pill faces. A licensing system should also require Chinese pill press manufacturers to fully disclose their production and distribution of presses and accompanying parts, allowing regulators to oversee these corporate operations more effectively.

China faces a challenging dilemma in balancing the needs of its manufacturing industries and the efficiency of port operations with its treaty obligations and ethical responsibilities to prevent the proliferation of fentanyl. China must decide how much it is willing to disrupt port operations to effectively surveil, scour, and investigate goods in transport for illicit pill press exportation. This includes cracking down on practices such as deliberately mislabeling packages and exporting to countries where the importation of unregistered pill presses is illegal. Enforcing comprehensive control over pill press distribution poses significant logistical challenges for port authorities, given the high volume of legally exported machinery per day and the difficulty in detecting deliberately mislabeled parts. China must also determine the amount of resources it will dedicate to these operations and the prosecution of violators.

Despite these concerns, China must strengthen its controls over pill press manufacturers and distributors while increasing oversight of online marketplaces and ports of exit. China should forward a licensing policy for pill press manufacturers, requiring them to meet explicit protocols and attain approval before distributing their pill presses domestically or exporting them abroad. Consequently, China can fulfill its global responsibilities to prevent the proliferation of illegal pill presses while preserving its status as a principal manufacturer of legal medical products.

### 4.3 International cooperation and border enforcement

Cartels and other criminal organizations have produced counterfeit fentanyl-laced pills since 2014.^30^ However, since 2019, the production of these counterfeit fentanyl-laced pills has accelerated, with fake pills being detected in all 50 U.S. states.^1^ The mortality rates from these counterfeit drugs will likely continue to rise unless decisive legislative and enforcement actions are taken at the state and federal levels in the U.S. This should include more substantial penalties for illegally distributing and using pill presses, enhanced surveillance and enforcement at ports of entry and international mail facilities, and cooperation among the U.S., China, and Mexico law enforcement in pill press control.

To disrupt the illegal distribution of pill presses, a multifaceted approach is needed, which includes increased border enforcement, domestic and international intelligence sharing, and advanced tracking methods. Most pill presses used for illegal purposes are manufactured in China, shipped to Mexico, and then used to tablet narcotics or smuggled into the U.S. Pill presses are also transported directly from China to the U.S. To disrupt these activities, U.S. law enforcement agencies at the federal, state, local, and tribal levels must work together to expand surveillance and enforcement efforts. For example, pill press tracking tactics should be implemented at border checkpoints. This involves attaching GPS devices to unregistered pill presses en route to their destinations, which enables investigators to track the delivery of pill presses and may provide reasonable cause to obtain search warrants at destination sites. Through tracking operations, law enforcement can locate and target previously unknown criminal enterprises.

The Chinese government should engage in bilateral and multilateral cooperation with the U.S. and Mexico, and work closely with regional and international organizations such as the United Nations Office on Drugs and Crime (UNODC) to advance the fight against counterfeit fentanyl-laced pill production. The U.S. and Mexico should urge China to increase surveillance of pill press exports, prosecute unscrupulous manufacturers and distributors selling to international actors, and investigate Chinese transnational criminal organizations (TCOs) selling pill presses directly to Mexican cartels and others with malicious intentions for use in narcotics manufacturing. Chinese port authorities and customs officials should also increase the surveillance and inspection of express consignment carrier (EEC) containers. Moreover, Chinese law enforcement should intensify the prosecution of those engaged in the illicit online sales of pill presses and molds.

In April 2023, Mexican President Manuel Lopez Obrador made a humanitarian request to China to strengthen controls over fentanyl shipped from China to Mexico and provide Mexico with regular accounts detailing fentanyl shipments between the two countries.^11^ This request could form the foundation for future anti-fentanyl collaboration, including strengthened control over pill press distribution.

Cooperation between the U.S., China, and Mexico is crucial to effectively control illicit pill press proliferation. However, language barriers can impede timely and efficient intelligence-gathering efforts. ChatGPT and other large language models (LLMs) can assist by providing instantaneous and highly accurate communication translations in various languages related to pill press activities.^33^ The ability to scan large amounts of foreign language communications (e.g., shipping manifests) and provide immediate and relatively accurate translations with indicators of potential illicit conduct can be valuable in cases involving foreign distribution operations.

China, the U.S., and Mexico should collaborate on creating a global coalition to establish a binding and complementary UN convention that explicitly regulates fentanyl and its analogues, fentanyl precursors and their analogues, pill presses, and dies and molds. Such a multilateral agreement could serve as a model for domestic legislation in countries seeking to control the production and distribution of these substances and related manufacturing equipment. However, countries may approach such a treaty with various concerns predicated upon the existence of domestic pill press manufacturing industries, whether it is importing presses, dies, molds, and associated parts for licit purposes, the size of ports of entry and exit likely requiring additional surveillance, and costs relating to increased enforcement and prosecution. These issues should be considered during the treaty formation process.

### 4.4 Limitations and future directions

A limitation of the study pertains to its exclusive focus on pill presses and counterfeit fentanyl-laced pills in illicit fentanyl production and control. Illicit fentanyl consumption encompasses a diverse array of modes, including powder inhalation, injection, polydrug combinations, and incorporation into consumables like candy. These substantial avenues of fentanyl use remain unconsidered within this review, particularly in relation to how other forms of fentanyl consumption may be impacted by increased pill press and counterfeit pill control.

An avenue for future research lies in the exploration of how alternative modes of illicit fentanyl production and consumption extending beyond the realm of pill presses and counterfeit pills may be influenced by increased pill press regulation and enforcement. Analyzing how fentanyl manufacturing and distribution strategies might adapt to compensate for increased pill press and counterfeit pill control would offer a deeper understanding of the strategies employed by illicit networks. By expanding the scope to encompass these additional modes of ingestion, future studies can contribute to a more expansive comprehension of the complexities surrounding the distribution and use of pill presses and counterfeit fentanyl-laced pills.

Another important consideration is the durability and adaptability of pill press production and the presses themselves. These devices can be utilized over extended periods, easily repaired, and shared or circulated within illicit networks. Pill presses can be manufactured and repaired within clandestine markets, further extending their availability and lifecycle. For example, 3D printing represents a significant avenue for future underground pill press, die, and mold production. Although this technology is in its early development stages and the pill presses made are of limited quality, pill presses can be printed that effectively produce counterfeit pills. Illicitly printed pill presses are challenging to trace and regulate and will likely be improved in the near term as the technology advances. This study does not extensively address the potential consequences of such control measures on the dynamics of certain underground operations or the adaptability of illicit networks.

The durable nature of pill presses and their potential to be manufactured and repaired in purely underground forums or other geographical areas represents an area for future study. By analyzing the potential pathways for criminals to exploit regulatory and enforcement changes, future researchers may illuminate the interplay between increased pill press regulation and control and the evolution of illicit activities.

## 5 Conclusions

The issue of pill press control has emerged as a critical factor in the fight against illicit fentanyl production. The U.S. and Chinese governments should establish comprehensive legislation that addresses nonexistent or weak penalties for pill press production, distribution, and possession with the intent to produce counterfeit fentanyl-laced pills. Additionally, illicit pill press possession and use should be considered an aggravating factor in sentencing rules. This would further incentivize law enforcement to enforce pill press laws against illegal tableting and pill press trafficking. By confiscating pill presses and incorporating them into larger drug-related cases, officials can better target the illicit manufacture and distribution of fentanyl.

Combating the devastating impact of counterfeit fentanyl-laced pills requires a multifaceted and comprehensive strategy. This approach should involve the U.S. and China establishing stronger legal provisions, enhancing law enforcement efforts, and engaging in international collaboration to reduce the distribution and use of illegal pill presses. By applying improved legislation and enforcement, the manufacture and distribution of deadly counterfeit fentanyl-laced pills can be controlled more effectively.

## Funding

No funding for this study was provided.

## Institutional review board statement

Not applicable.

## Ethics statement

Not applicable.

## Declaration of Competing Interest

The author has no competing interests that could influence the work reported in this paper.

## References

[1] Legislative Analysis and Public Policy Association (2021). Pill Presses: Summary of State Laws. https://www.safemedicines.org/wp-content/uploads/2019/09/2021-Pill-Presses-Summary-of-State-Laws.pdf.

[2] United States Drug Enforcement Administration (2022). https://www.dea.gov/alert/dea-laboratory-testing-reveals-6-out-10-fentanyl-laced-fake-prescription-pills-now-contain.

[3] United States Drug Enforcement Administration (2021). https://www.dea.gov/press-releases/2021/09/30/dea-seizes-historic-amounts-deadly-fentanyl-laced-fake-pills-public.

[4] Ganim S. (2017). Pill presses for counterfeit drugs seized in record numbers. CNN.

[5] U.S. Department of Treasury (2023). https://home.treasury.gov/news/press-releases/jy1507.

[6] Felbab-Brown V. (2022). China and synthetic drugs control: fentanyl, methamphetamines, and precursors. Brookings..

[7] United States Drug Enforcement Administration (2020). https://www.dea.gov/documents/2020/2020-03/2020-03-06/fentanyl-flow-united-states.

[8] The White House (2023). https://www.whitehouse.gov/briefing-room/statements-releases/2023/04/11/fact-sheet-biden-harris-administration-announces-strengthened-approach-to-crack-down-on-illicit-fentanyl-supply-chains/.

[9] Associated Press (2022). https://apnews.com/article/colorado-united-states-mexico-arizona-yuma-a872d9c301bed59d8f01e95f8afcb667.

[10] United States Department of Justice (2017). https://www.justice.gov/usao-ct/pr/west-hartford-man-admits-importing-pill-press-china-make-fake-oxycodone-pills-containing.

[11] Reuters (2023). Mexico asks China for help on fentanyl and slams U.S. critics. https://www.nbcnews.com/news/world/mexico-asks-china-help-fentanyl-slams-us-critics-rcna78269.

[12] Kamp J., Córdoba J.D., Wernau J. (2022). Inside the Mexican cartels that rule fentanyl. Wall Street Journal.

[13] Sherman C., Stevenson M. (2023). “El Chapo” sons charged with smuggling cheap fentanyl to US. Associated Press.

[14] United States Drug Enforcement Administration (2022). https://www.dea.gov/press-releases/2022/08/30/dea-warns-brightly-colored-fentanyl-used-target-young-americans.

[15] Drug Enforcement Administration Facts About Fentanyl. https://www.dea.gov/resources/facts-about-fentanyl.

[16] United States Drug Enforcement Administration Fentanyl Awareness. https://www.dea.gov/fentanylawareness.

[17] National Institutes of Health Drug Overdose Death Rates. https://nida.nih.gov/research-topics/trends-statistics/overdose-death-rates.

[18] Centers for Disease Control and Prevention Fentanyl Facts. https://www.cdc.gov/stopoverdose/fentanyl/index.html.

[19] Kariisa M., O’Donnell J., Kumar S., Mattson C.L., Goldberger B.A. (2023). Illicitly manufactured fentanyl–involved overdose deaths with detected xylazine — United States, January 2019–June 2022. MMWR Morb Mortal Wkly Rep.

[20] The State Council Information Office of the People’s Republic of China The Entire Category of Fentanyl-like Substances will be Listed. http://www.scio.gov.cn/34473/34474/Document/1651166/1651166.htm.

[21] International Narcotics Control Board (2023). Materials and equipment. https://www.incb.org/incb/en/precursors/materials-and-equipment.html.

[22] United States Drug Enforcement Administration Two Marysville, Washington residents arrested following discovery of fentanyl pill manufacturing lab. https://www.justice.gov/usao-wdwa/pr/two-marysville-washington-residents-arrested-following-discovery-fentanyl-pill.

[23] Medicines Safe (2019). A Joint Project by the National Association of Boards of Pharmacy, the National Association of Drug Diversion Investigators, and the Partnership for Safe Medicines.

[24] 21 U.S.C. § 830 Regulation of Listed Chemicals and Certain Machines. https://www.law.cornell.edu/uscode/text/21/830.

[25] 21 U.S.C. § 843 Prohibited Acts C. https://www.law.cornell.edu/uscode/text/21/843.

[26] 15 U.S.C. § 1116 Injunctive Relief. https://www.law.cornell.edu/uscode/text/15/1116.

[27] 21 U.S.C. § 333 Penalties. https://www.law.cornell.edu/uscode/text/21/333.

[28] Florida Legislature (2022). http://www.leg.state.fl.us/statutes/index.cfm?App_mode=Display_Statute&URL=0800-0899/0893/Sections/0893.147.html.

[29] Kustoff D. (2023). Press Release from the Congressional Office of David Kustoff.

[30] United States Drug Enforcement Administration Counterfeit prescription pills containing fentanyls: A global threat. https://www.dea.gov/sites/default/files/docs/Counterfeit%2520Prescription%2520Pills.pdf.

[31] General Administration of Customs PR of C. Customs law of the People’s Republic of China http://english.customs.gov.cn/statics/644dcaee-ca91-483a-86f4-bdc23695e3c3.html.

[32] United Nations Office on Drugs and Crime (1988). https://www.incb.org/documents/PRECURSORS/1988_CONVENTION/1988Convention_E.pdf.

[33] Dreibelbis E. (2023). Google Translate vs. ChatGPT: Which one is the best language translator?. PC Magazine.

